# Reframing Emergency Medical Service in the context of chronic non-communicable disease and palliative care

**DOI:** 10.4102/phcfm.v18i1.5206

**Published:** 2026-03-31

**Authors:** Linley A. Holmes, Elizabeth Gwyther, Shannon Odell

**Affiliations:** 1 Division of Interdisciplinary Palliative Care and Medicine, Department of Family, Community and Emergency Care, Faculty of Health Sciences, University of Cape Town, Cape Town, South Africa

**Keywords:** EMS, palliative care, chronic non-communicable disease, symptoms, clinical interventions, caregivers, patient-centred care, South Africa

## Abstract

**Background:**

Emergency Medical Service (EMS) is often the initial primary contact for patients with non-communicable disease (NCD) with symptom exacerbation. EMS personnel are not trained to manage patients requiring this type of care, or recognise the need for palliative care or expert consultation.

**Aim:**

To describe how EMS’ are often responsible for managing patients with NCDs and symptom exacerbation and to assess whether palliative care and support by EMS in the out-of-hospital sector should be considered.

**Setting:**

The study took place in the southern peninsula of Cape Town, including urban, suburban and rural communities, including the informal housing community.

**Methods:**

This was a retrospective descriptive analysis of de-identified patient report forms of adult patients (over 18 years old) attended to by a private EMS between January 2019 and April 2019. The patients included in the research met the inclusion criteria, identifying them as possibly requiring palliative care assistance because of the symptom exacerbation of their NCDs.

**Results:**

Of the 283 patients many had more than one NCD, and more than one of the primary symptoms of pain, shortness of breath, cognitive changes, nausea and vomiting simultaneously. The majority of these patients were likely to go to the hospital with ambulance transport, and frequently, there was no appropriate clinical intervention by paramedics. Clinical advice was seldom sought by paramedics for patients with symptom exacerbation related to their NCDs, with the data indicating that palliative care access for patients was minimal.

**Conclusion:**

Palliative care is a necessary approach to patient-centred care, with EMS being an available resource to assist with this approach. There is a necessity for improved communication and support between palliative care resources and EMS to mitigate inappropriate transport of these patients to already over-burdened emergency departments, and to improve home care by EMS.

**Contribution:**

This study highlights the need for appropriate palliative care support by EMS for patients with chronic disease and symptom exacerbation.

## Background

The incidence of non-communicable diseases (NCDs), such as cardiovascular and respiratory diseases and cancer, is rising globally, putting strain on healthcare systems focused on curative care.^1,2^ Access to healthcare is hindered by poverty, urbanisation and high disease burdens, particularly in central and sub-Saharan Africa, contributing to 41 million deaths annually and worsening socio-economic inequalities.^3^ Limited healthcare and lack of palliative care exacerbate morbidity and mortality, as seen in South Africa.^4,5^ Addressing NCDs requires proactive, integrated chronic disease care both in and outside hospitals,^5^ including prevention, diagnosis and treatment while considering socio-economic factors.^6^ Preventive care, interdisciplinary approaches, and integrated acute, primary, and palliative care are essential.^3^ However, many NCD patients still lack access to comprehensive palliative care, impeding holistic management of their needs.^3,4,5,6,8^

Palliative care in South Africa began with the establishment of hospices in the 1980s, with the formation of the Hospice Palliative Care Association (HPCA) in 1987, now the Association of Palliative Care Centres of South Africa (APCC).^9^ Palliative care became more integrated into the healthcare sector in response to the human immunodeficiency virus (HIV) and acquired immunodeficiency syndrome (AIDS) epidemic. In 2017, the National Policy Framework and Strategy for Palliative Care (NPFSPC) was approved, highlighting the necessity of resource allocation across all healthcare levels to manage chronic diseases.^8^ Global challenges such as poor communication about prognosis and death, lack of provider awareness about palliative care, ineffective assessment of functional decline, and delayed initiation of palliative care have resulted in the need for a consistent, ‘tool-based’ approach.^3,7,9,10^ Hence, the South African Supportive and Palliative Care Indicator Tool (SPICT) model (SPICT^SA^) was introduced to enable earlier identification of patients requiring palliative care.^10^

The past decade has seen a rise in the number of patients with NCDs seeking emergency services because of disease exacerbations, challenging Emergency Medical Services (EMSs) and prompting calls for EMS reform.^11,12^ Demographic shifts and increasing healthcare demands have lengthened EMS response times and caused overcrowding of Emergency Departments (EDs), necessitating improved training and interprofessional collaboration.^12^ While specialised EMS roles could reduce emergency admissions, EMS scope and training are limited in South Africa. Community EMSs in Denmark and Canada have shown success in managing chronic diseases and reducing hospitalisations.^13,14^ Research from Australia and France underscores the need for palliative care education for EMSs, emphasising patient-centred care and cooperation between healthcare disciplines such as EMS, palliative care and general practitioners (GPs).^15,16,17^ Programmes such as ‘EMSs Providing Palliative Care at Home’ in Canada have yielded positive outcomes, highlighting a need for policy changes.^14,17^ Integration of EMS and palliative care can enhance care access, reduce hospitalisations, and alleviate EMS burdens, particularly in LMICs, as noted by Gage et al.^18^ Emergency care traditionally centres on life-saving interventions, but increasing end-of-life (EOL) care needs, often involving directives like Do Not Attempt Resuscitation (DNARs) and Medical Orders for Life-Sustaining Treatment (MOLSTs), require greater EMS education and clearer protocols.^19^ While most EMSs receive training on these directives, many seek further education on legal and EOL care issues, emphasising the importance of early public education, better residential care training and improved EMS protocols. Lord et al. comment that EMSs are bound by standard operating procedures and scope of practice, and are often required to assess and transport patients to an emergency centre, independent of their medical history. This results in few patients being managed at home.^15,20^ This is reiterated in the ‘South African paramedics perspectives on pre-hospital palliative care’ publication, describing a topic that is becoming more relevant and challenging to paramedics in the out-of-hospital environment.^21^

There was a clear lack of data on South African EMS involvement in palliative care and chronic disease management, which contrasts with international evidence. This indicates potential for scalable programmes in South Africa, which can potentially be modelled on the Chronic Care Model (CCM),^22^ the Early Palliative Intervention Care model (EPIC),^18^ and international training collaborative like Learning Essential Approaches to Palliative Care (LEAP) (Canada).^23^ The potential alignment of global EMS and palliative care models is potentially going to be realised with recent Health Professionals Council of South Africa (HPCSA) updates^24^ offering a pathway to more compassionate care through the introduction of Clinical Decision Support Tools (CDST) for emergency care providers.^22,25^

The South African EMS context has seen little focus on palliative care, but recent research highlights its growing relevance.^18,21^ South Africa has both private (medical aid-supported or for-profit services) and public sector emergency services (not-for-profit). Both of these sectors run a combination of ambulances and response vehicles managed by EMS with varying scopes of practice from basic life support to advanced emergency care.

### Aim

This study aims to outline the impact of NCDs on EMS in South Africa and explore EMS’ understanding and application of palliative care. It focuses on NCD patients in South Africa with acute symptoms, whether they were transported to hospital via ambulance, clinical management, and the need for better out-of-hospital palliative care through education, collaboration and service provision.

## Research methods and design

### Setting

The southern peninsula of Cape Town, which consists of urban, suburban and rural communities, including an expanding informal housing community. This area is covered by an in situ private EMS company (as well as by Metro EMS and other more remote private EMS companies) that provides support to both members and the public as needed.

### Design

This was an observational, retrospective, descriptive analysis of adult patient records of a private EMS service in the southern peninsula of Cape Town, also providing a service for many public sector callouts in this area.

### Data extraction

This study reviewed anonymous patient report forms (PRFs) for individuals aged 18 years or older who used EMS service between 01 January 2019 and 30 April 2019. All adults (both male, female and other) residing within the emergency service’s area, including both members and non-members, were included.

Patient Report Forms were extracted with management’s permission and assessed for inclusion. A data extraction sheet was developed by the researcher, informed by literature reviews and discussions with both EMS personnel and palliative care professionals. Supervisors (Liz Gwyther and Shannon Odell) reviewed and revised the sheet, which incorporated many parameters from the researcher’s experience. Variables included the primary reasons for EMS callout and symptoms recorded by EMS (shortness of breath [SOB], pain, nausea, cardiac arrest and cognitive changes); these symptoms and associated NCDs (cancer, heart disease, dementia, lung disease, diabetes and renal failure), as well as EMS interventions for these symptoms. Care plans such as doctor referral letters, advanced care plans, living wills and hospital appointment letters were included in the variables recorded, while patient transport and symptom management decisions, such as transport to the ED, no transport to hospital (or refusal of care), or no interventions for associated symptoms, were captured.

Data capture allowed for trend analysis, following ethical guidelines (DoH 2015). Data access, which was limited by COVID-19 restrictions (the researcher was unable to visit the EMS site to access the PRFs), began in June 2020. Patient report forms from January 2019 to April 2019 were manually reviewed and entered into Google Forms. All PRFs were paper-based and securely handled by the researcher Linley A. Holmes without confidentiality breaches.

Though standardised PRFs were used, variations in EMS records created inconsistencies. Cases were included if they referenced terms related to chronic diseases, palliative care or EOL conditions. Data were backed up on a secure server and anonymised. Access to patient data was restricted to on-site use, with strict confidentiality and security measures followed throughout.

Exclusion criteria included the PRFs of minors (any person under the age of 18 years); PRFs not including any of the key points were excluded from the research, and the PRFs of patients that had reported having a communicable disease, in the absence of NCD.

### Data analysis

Patient report forms were analysed, and data variables were extracted using Google Docs and Google Sheets (Google LLC, Mountain View, CA, United States [US]) based on the data collection tool and inclusion/exclusion criteria, then placed in Microsoft Excel (Microsoft Corporation, Redmond, WA, US). Python (Python Software Foundation, Wilmington, DE, US) and Jupyter Notebook (Project Jupyter, https://jupyter.org) were subsequently used for data preparation, cleaning, statistical calculations and visualisation.

Data collected via the data extraction sheet described in the data collection process above were exported to a comma-separated value (.csv) file using Microsoft Excel. A separate .csv file was created, only including data that met the eligibility criteria of the study. This was called the ‘valid calls’ data set. Each variable in the ‘valid calls’ data set was analysed, and unique entries for each variable were tabulated and counted. This provided a data frame to use for statistical analysis and visualisation. The data extracted were analysed and are presented using tables and graphs as descriptive tools.

### Sample size

A sample size of *N* = 246 (PRFs) was calculated to provide sufficient data for reliability. A simple sampling method was used to extract relevant patient records (as per inclusion criteria) between the dates of 01 January 2019 and 30 April 2019.

The following sample size calculation was used to predict how many PRFs would be needed to prove reliability.

Sample size for a single proportion:

**Table:** 

• Enter expected proportion (p) %	20
• Enter precision (d) %	5
• Required sample size (n)	246

### Ethical considerations

Ethics approval was granted by the University of Cape Town Health Research Ethics Committee in writing. Study approval number: HREC REF:176/2020.

## Results

During the study from 03 January 2019 to 30 April 2019, the above-mentioned EMS responded to 1572 callouts, with 283 (18%) meeting the research criteria. The study assessed patient-EMS consultations, EMS approach to NCDs, clinical decision-making, EMS consultations with alternate clinical practitioners, and support for patients or carers benefiting from palliative care programmes.

Cancer was the most prevalent disease recorded in 108 (29%) cases, followed by chronic heart disease (*n* = 86, 23%), dementia (*n* = 60, 16%), chronic lung disease (*n* = 56, 15%), diabetes (*n* = 37, 10%) and renal failure (*n* = 26, 7%). The total number of NCDs (*n* = 374) recorded exceeded the patient numbers (*n* = 283) but were included in the study as some of the patients had more than one NCD.

**FIGURE 1:**
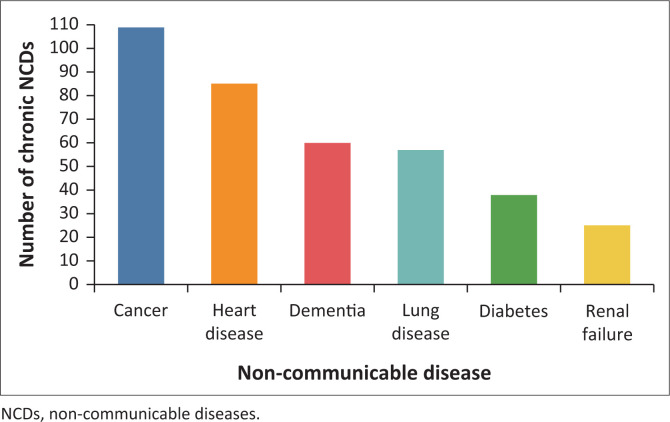
Frequency of chronic non-communicable diseases recorded in patient report forms from 283 eligible Emergency Medical Service callouts.

**FIGURE 2:**
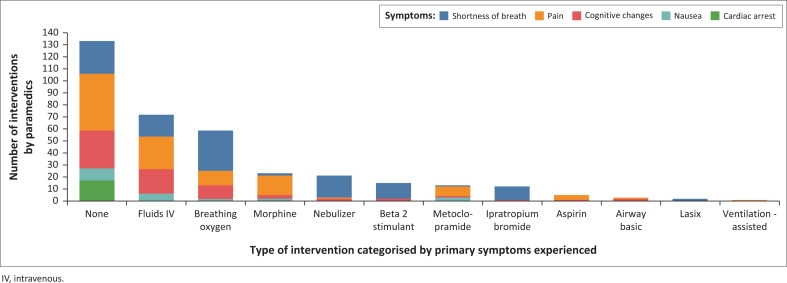
Number and type of interventions performed by Emergency Medical Service, categorised by the primary symptom experienced by the patient.

Data analysis shows that EMSs were called out to symptoms (Table 1) often associated with identified chronic NCDs (Figure 1), with EMS often not performing interventions for primary patient symptoms. For patients with SOB, common interventions were oxygen, nebulisation, and intravenous (IV) fluids, though no intervention was recorded in 22% of cases, possibly because adequate oxygen levels (AHA and HPCSA guidelines recommend maintaining oxygen saturation between 92% and 98%). Pain went untreated in 40% of cases, with IV fluids, morphine, and oxygen being the most common interventions when used. Cognitive changes lacked interventions in 42% of cases, with IV fluids and oxygen being the most common treatments. For nausea and vomiting (N&V), 43% received no intervention, with IV fluids and antiemetics rarely provided. None of the 17 cardiac arrest patients received resuscitation or intervention, as they were recorded as deceased (P4). While cardiac arrest was noted in 17 cases, 20 patients were recorded as P4, with three PRFs not indicating particular patient symptoms prior to cardiac arrest (Figure 2 and Table 2).

**TABLE 1:** Primary reasons (matching study criteria) for callouts recorded and primary symptoms recorded by Emergency Medical Service (percentage broken down by primary reason and symptoms).

Reason	Primary reasons for callouts recorded		Primary symptoms recorded by EMS	
	%	*n*	%	*n*
Shortness of breath	43	81	30	88
Pain	35	66	34	100
Nausea	10	19	7	22
Cardiac arrest	6	11	7	20
Cognitive changes	5	10	23	67

**Grand total**	**100**	**187**	**100**	**297**

**TABLE 2:** Number of specific interventions performed by Emergency Medical Service for each of the most prevalent patient symptoms.

Symptoms	Intervention	Number of interventions by EMS
Shortness of breath	None	27
	Fluids IV	18
	Oxygen	33
	Morphine	2
	Nebuliser	18
	Beta 2 stimulant	13
	Metoclopramide	1
	Ipratropium bromide	11
	Furosemide	2
	Total	125
Pain	None	47
	Fluids IV	27
	Oxygen	12
	Morphine	16
	Nebuliser	1
	Metoclopramide	8
	Aspirin	4
	Airway basic	1
	Ventilation – assisted	1
	Total	117
Cognitive changes	None	31
	Fluids IV	20
	Oxygen	11
	Morphine	3
	Nebuliser	2
	Beta 2 stimulant	2
	Metoclopramide	1
	Ipratropium bromide	1
	Aspirin	1
	Airway basic	2
	Total	74
Nausea	None	10
	Fluids IV	6
	Oxygen	2
	Morphine	2
	Metoclopramide	3
	Total	23
Cardiac arrest	None	17
	Total	17

**Grand total**	-	**356**

EMS, Emergency Medical Service; IV, intravenous.

Of the 192 patients transported by ambulance to the hospital, 129 (67%) of the patients went to the ED. Of the patients transported by ambulance, 23 (12%) went to a palliative care unit, 15 (8%) went home from a facility, 12 (6%) went to frail care, 11 (6%) went to a hospital ward, and 2 (1%) went to a clinic. The location for the remaining patients were not transported by ambulance.

**FIGURE 3:**
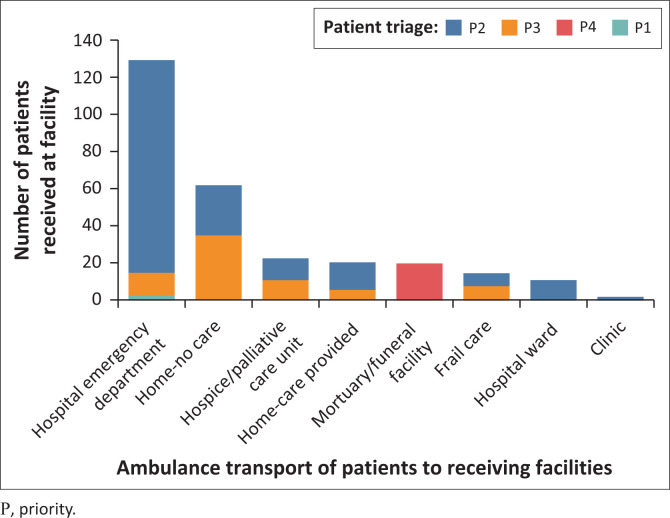
Patients transported by Emergency Medical Service to different receiving facilities.

A total of 192 patients were transported by ambulance to various facilities as indicated in Figure 3 and Table 3, with 157 primary symptoms recorded for the 129 patients transported to an ED (Table 4). Of these symptoms, pain ranked highest, with SOB, cognitive changes and N&V being the other more prominent symptoms recorded. Many patients had multiple symptoms. Among the 91 patients not transported by ambulance, 71 primary symptoms of pain, SOB, cognitive changes, N&V and cardiac arrest were recorded. For patients receiving no treatment, many had pain, cognitive changes and SOB, with a smaller percentage experiencing N&V. None of the 17 patients in cardiac arrest received intervention (Table 4).

**TABLE 3:** Triaged patients opting for ambulance transport, no transport, or private transport, to a receiving facility.

Patient triage	Transport								Total	
	Ambulance		None		Private		Mortuary van		*n*	%
	*n*	%	*n*	%	*n*	%	*n*	%		
P2	154	82	30	16	3	2	0	0	**187**	**100**
P3	35	48	37	51	1	1	0	0	**73**	**100**
P4	0	0	17	85	0	0	3	15	**20**	**100**
P1	3	100	0	0	0	0	0	0	**3**	**100**

**Total**	**192**	**68**	**84**	**30**	**4**	**1**	**3**	**1**	**283**	-

P, priority.

**TABLE 4:** Symptom prevalence among patients transported to the emergency departments and those not transported by ambulance.

Symptoms	Symptoms recorded of patients transported to EDs by EMS		Symptoms recorded of patients not transported by ambulance	
	*n*	%	*n*	%
Pain	36	57	28	20
Shortness of breath	33	52	28	20
Cognitive changes	21	33	14	10
Nausea	10	15	6	4
Cardiac arrest	N/A	-	24	17

**Grand total**	**100**	**157**	**100**	**71**

ED, Emergency Departments; EMS, Emergency Medical Service.

In the 283 PRFs assessed, there was a paucity of living wills and advanced care planning (ACP), with doctor referral letters making up only 6.4% of clinical, patient or caregiver communication (Table 5).

**TABLE 5:** Patients with care plans and associated triage scores.

Care plan	Patient triage	Patients	
		*n*	%
None	P2	172	92
	P3	63	86
	P4	19	95
	P1	3	100
	Total	257	91
Doctor’s referral letter	P2	9	5
	P3	9	12
	Total	18	6
Advanced care plan	P2	1	1
	P3	1	1
	P4	1	5
	Total	3	1
Living will	P2	3	2
	Total	3	1
Hospital appointment card	P2	2	1
	Total	2	1

**Gr and total**	-	**283**	**100**

P, priority.

Among the 283 patients, many had more than one NCD and experienced multiple primary symptoms (pain, SOB, cognitive changes, N&V). Most patients were triaged as low priority, and often no EMS intervention was provided, with explanations for treatments being rare. Clinical consultation was seldom sought, and care plans were scarce.

These findings raise questions about why NCD patients call EMS, the reasoning behind hospital transport decisions, and whether they receive appropriate care by EMS at primary contact, and whether palliative care for symptom exacerbation in chronic disease is applied in the ED.

## Discussion

The data found that the EMS service examined in this research responded to a significant proportion of patients with NCDs experiencing an acute exacerbation of their disease. Patients experiencing uncomfortable symptoms of SOB, pain, N&V and cognitive changes prompted a request for EMS support. It is evident that patients were frequently transported to EDs by EMS, but received inconsistent, or no symptom management prior to, or during transport. This is consistent with healthcare models, including those of EMS, that have been based on providing curative care.^12,17,18^ The consideration here is that EMS is often utilised as a transport system rather than providing primary care for patients with NCDs at home. This emphasises the need for EMS to have training with clinical guidelines and clinical support structures for interventional patient care to be initiated at home. Thus, transportation to an ED could be avoided in many cases. In recognition of a changing disease profile with NCDs on the rise, and the need to provide appropriate care to the general patient population, EMS in parts of the world are revising their historical culture of care.^24^

As patients seldom have any form of ACP or specialised consultant contacts, there is often little support for these patients and their caregivers, and EMS becomes the first port of call for assistance.^13^ Similarly, EMSs are often disempowered when making patient care and transport decisions with no guidance or consultant support and are forced to transport patients to the hospital inappropriately. The reasons for this are linked to the scope of clinical practice, EMS protocols and procedures, lack of support structures such as palliative care networks and home care services, and ineffective discharge plans.^26^ Globally, there seems to be a lack of palliative care and NCD symptom management education in undergraduate syllabi for medical students with similar correlation to EMS Paramedicine,^27,28^ and a lack of clinical support structures within the community or available around the clock accessible care.^15,18,19,20,21,26,29^ In line with this, EMS systems and tertiary facilities worldwide are researching, initiating, or have already included palliative care training in their core curricula and service infrastructure.^14,15,16,17,22,25,29,30,31^

Diaz et al. found that patients with chronic disease are often brought to the ED, as there are no other perceived options for their symptom management, even though they may not fit into an acute care or triage profile.^30^ However, the point is made that this type of patient flow could be diverted away from emergency centres, encouraging patient care rather at respite care facilities.^25,26^ Acute injury or illness is often managed through an algorithmic approach in the EMS or ED space.^22,26^ This can differ when managing chronic disease exacerbations. Algorithms, with emergency interventions, may not be appropriate for the patient with an NCD.^30,31,32^ These patients rely on care that is based on disease trajectory, considers medications, prior symptom exacerbations and hospital visits, and considers multifactorial influences such as psychosocial and spiritual well-being. The relevance here is that emergency services often fulfil a ‘transport’ service rather than a service providing clinical or supportive care, evident by the fact that many of the patients transported by the private EMS received no care, or clinically unnecessary care, with most patient handovers occurring at the ED.^21,25^

With the advent of SPICT and SPICTSA,^10^ there is a higher likelihood that with education and training, EMSs and ED staff may more easily identify patients with NCDs requiring a different care approach and possibly palliative care assistance.

The challenges for EMSs managing patients with chronic disease, potentially those who could benefit from a palliative care programme within EMS, are complex. This is because of the various EMS scopes of practice, from basic life support (BLS) through strata of practice, eventually culminating in three different levels of advanced life support (ALS). These scopes of practice, under the clinical practice guidelines of HPCSA, dictate the decision-making abilities of each practitioner, and what interventions that practitioner can use. The scope of practice and recommendations for practitioners have recently been adjusted through a Clinical Decision-Making Tool focusing on recognition and guidelines for managing palliative care patients.^22,25^

The traditional role of EMSs has been primarily viewed as a transportation system, focusing on rapid response times and swift delivery of patients to healthcare facilities. This model often emphasises metrics such as call times and transport efficiency over comprehensive patient care at home. Consequently, EMS efficacy is frequently measured by these operational parameters, aligning with historical approaches that prioritise hospital-based interventions, such as those requiring operating theatres, for definitive patient management.^33^

Moreover, the focus on rapid transportation over in-home care has been identified as a limitation in the current EMS model. Integrating EMS with home health services has been proposed as a strategy to improve patient outcomes, particularly for those with chronic conditions who may benefit from receiving care in their home environment. A report by the National Association of Emergency Medical Technicians discusses how EMS-based Mobile Integrated Healthcare (MIH) programmes can complement home health services, suggesting that such collaborations can fill gaps in the care delivery model and provide more patient-centred care.^34^

These insights underscore the need for a paradigm shift in EMS operations, moving from a transport-centric model to one that prioritises effective patient care, seamless information transfer and integration with broader healthcare services. A joint decision in these cases would be far more effective and supportive of the patient’s wishes and the space in which they convalesce or die.^9,16,27,28,31,33,34^

Therefore, to address the barriers in EMS and palliative care, EMS should be integrated into national and international palliative care policies. A proposed Public Health Palliative Care approach such as the Australian ‘Healthy End of Life Program’ (HELP) and ‘Early Palliation through Integrated Care’ (EPIC) aims to link EMS to formal and informal care networks, ensuring equitable access to underserved groups, reducing hospital admissions, and supporting home-based deaths.^14,23,24,27,34^ Overcoming cultural barriers requires evolving EMS personnel’s perceptions through education and training. Additionally, societal attitudes towards EMS’s role in community palliative care must shift via campaigns promoting death literacy. Research should explore alternative referral pathways, including telehealth and community-based models, to integrate EMS into palliative care systems and enhance care continuity.^28,29,35^

Presently, a collaborative project, led by the Division of Emergency Medicine in the Department of Family, Community and Emergency Medicine, University of Cape Town, is working towards a telehealth system aimed at supporting EMS caring for patients with cancer. This pilot will evaluate an interdisciplinary consultation model to enhance EMS-delivered palliative care, whether at home or providing access to appropriate facilities. This is one of the many approaches that indicate a recognition in the out-of-hospital sector that not all patients fall under the curative algorithm of EMS care but require a different approach that focuses on patient quality of life and interdisciplinary healthcare provision.^35,36,37^

### Limitations

Although the period of data researched and presented was only over 4 months (01 January 2019 to 30 April 2019), the study highlights gaps in EMS data related to patients with NCDs who may require palliative care, noting that symptoms and keywords merely estimate potential need rather than accurately identifying current demand. Structural deficiencies in PRFs, such as the omission of risk factors like smoking, likely lead to underdiagnoses of conditions such as chronic obstructive pulmonary disease and emphasise the need for repeat call-out data for better insight; this is also a limitation of the retrospective research design.

The timing and restrictions on data collection presented by COVID-19 influenced the timing and consistency of data collection, which could have resulted in inaccurate and missing data.

## Conclusion

Palliative care is critical for managing NCDs and requires healthcare systems to adapt in alignment with WHO goals.^3,7,9^ While EMSs are integral to NCD care, this study found that 20% of EMS patients had NCDs, yet EMSs often provided inadequate care, with the possibility of insufficient training, potentially limited understanding of NCDs, and access to home care support or palliative care networks. Many patients were transported to hospitals without clear treatment plans, reflecting gaps in transitional care and EMS management of NCD symptoms. The study recommends integrating palliative care training into EMS education, developing collaborative care networks among EMS, GPs, and allied health professionals, and emphasising ACP throughout disease trajectories. Such measures aim to enhance continuity of care, reduce hospitalisations, and improve patients’ quality of life while supporting caregivers.^35,36,37,38^
